# Smoking and the Risk of Upper Aero Digestive Tract Cancers for Men and Women in the Asia-Pacific Region

**DOI:** 10.3390/ijerph6041358

**Published:** 2009-04-03

**Authors:** Alireza Ansary-Moghaddam, Alexandra Martiniuk, Tai-Hing Lam, Konrad Jamrozik, Akiko Tamakoshi, Xianghua Fang, Il Suh, Federica Barzi, Rachel Huxley, Mark Woodward

**Affiliations:** The Asia Pacific Cohort Studies Collaboration, Mount Sinai Medical School, Box 1087, One Gustave L. Levy Place, New York, NY 10029, USA

**Keywords:** Cancer of the pharynx, cancer of the larynx, cancer of the esophagus, smoking, alcohol

## Abstract

Although smoking is an established causal factor for upper aero digestive tract cancer (UADTC), most of the evidence originates from the West. Thus, we analysed data from 455,409 subjects in the Asia Pacific Cohort Studies Collaboration. Over a median of around six years follow-up, 371 deaths from UADTC were observed. The hazard ratio (95% confidence interval) for current smokers, compared with those who had never smoked, was 2.36 (1.76 – 3.16), adjusted for age and alcohol drinking. Tobacco control policies are urgently required in Asia to prevent millions of deaths from UADTC that smoking will otherwise cause.

## 1. Introduction

Upper aero-digestive tract (lip, oral cavity, pharynx, esophagus, larynx) cancers are an important global problem, particularly in developing countries. Since three-quarters of the nearly one million cases of upper aero-digestive tract cancer (UADTC) occurring annually are seen in developing countries, UADTC ranks among the five most common cancers in both men and women in these settings [1]. Whilst there is considerable variation in survival across the constituent cancers in the UADTC group, on average UADTC has one of the lowest figures for five-year survival of all cancers, with a range from 10% to 65% [2–4].

Cigarette smoking is a recognised causal factor for UADTC [2–6]. The excess risk of UADTC associated with smoking varies depending on the subtype of cancer and the intensity and duration of smoking [7]. Alcohol drinking is the other main risk factor [3,4,6,7], having been reported to increase the risk of developing UADTC among non-smokers by more than four times [7]. Combined, these two risk factors have been estimated to increase the risk of these cancers by more than twelve times compared with individuals who neither smoke nor drink alcohol [7].

Most of the data examining the aetiological role of smoking on UADTC are derived from studies in Western countries, with few data from populations in eastern and south-eastern Asia (here referred to as “Asia”, for brevity). It is not known whether the effects of smoking on the risk of UADTC differ substantially between Asian and non-Asian populations. Region-specific information as to the magnitude of risks associated with these risk factors would assist public health specialists and policy makers in Asia. This is especially relevant, given that the consumption of tobacco in many countries in Asia is very high amongst men [8–10], whilst there is an upwards trend in smoking amongst young women in some of these countries [11]. Furthermore, in countries such as China, where more than 60% of adult men smoke, the risks associated with smoking are largely unrecognised [9].

The current paper uses data from the largest collaborative study in the region, the Asia Pacific Cohort Studies Collaboration (APCSC), to quantify the risk of mortality from UADTC associated with smoking, taking account of potential confounding and modifying effects from alcohol, in populations of the Asia-Pacific region.

### 1.1. Methods

Details of study identification, data collection and event verification for studies in the APCSC are described elsewhere [12]. Briefly, studies were included if they had continued follow-up for at least 5,000 person-years and had recorded vital status at the end of follow-up. Studies were excluded from APCSC if entry to that study was dependent upon having a particular medical condition; for this current paper only, studies were additionally excluded if they did not record deaths due to UADTC. We restricted our analyses to individuals aged 20 or more years at enrolment. Mortality was classified according to the 9th Revision of the International Classification of Diseases (ICD): UADTC was selected as codes 140–149 (lips, tongue and pharynx; this includes: malignant neoplasms of lip, tongue, major salivary glands, gum, floor of mouth, other and unspecified parts of mouth, oropharynx, nasopharynx, hypopharynx, other and ill-defined sites within the lip), 150 (esophagus), and 161 (larynx). Studies were classified as Asian if their participants were recruited from mainland China, Hong Kong, Japan, Korea, Singapore, Taiwan or Thailand; and ANZ if from Australia or New Zealand.

All data on cigarette smoking and alcohol drinking were based on self-report at the time of entry into each of the included studies (‘baseline’). Data recorded on smoking included the number of cigarettes currently smoked per day, categorized here into groups of < 20 and ≥ 20 (20 cigarettes corresponds to one standard pack), and whether or not non-smokers were former smokers. Alcohol was only recorded as current drinking or not. Participants were included in this study only if they reported their current and past smoking and current alcohol drinking at baseline. In APCSC, 94% of the 600,445 participants recorded their current smoking status, 92% recorded their current drinking status and 91% reported both.

Cox proportional hazard models, stratified by study and sex, and adjusted for age, with and without additional adjustment for alcohol, were used to estimate hazard ratios (HRs) associated with smoking (current *v* never, and dose-response) for death due to UADTC and its subtypes. We also estimated HRs for overall UADTC by sex, geographical area (Asia/ANZ) and alcohol subgroups, and tested for effect modification by including interaction terms in the Cox models [13]. In order to avoid reporting unreliable results when the number of events was small, estimates of relative risk for UADTC subtypes are not reported for these subgroups. The effect of quitting smoking was estimated through HRs comparing ex-smokers with current smokers. All analyses used individual participant data.

The population attributable risk for mortality from UADTC associated with smoking were calculated for each country, for both males and females, using previously published recent prevalence estimates of the prevalence of smoking [10] and the formula [13]: PAR = [P (RR-1)]/ [1 + P (RR-1)]. Here, the RR was that found from APCSC.

## 2. Results and Discussion

Of the 44 studies in APCSC, 26 recorded current and past smoking and current drinking at baseline as well as deaths from UADTC. These 26 studies (19 from Asia) included 455,409 men and women (87% from Asia; 32% female) (Table 1). There were large differences in the percentage of smokers between the regions and sexes: 23% and 20% of men and women in ANZ and 59% and 5% of men and women in Asia, respectively. On average, smokers in ANZ smoked more cigarettes per day than those in Asia: 18 and 15 cigarettes per day in ANZ for men and women, respectively, compared with 15 and 11 in Asia. The percentage of individuals who reported that they consumed alcohol at study baseline also differed by region and by sex: 84% in men and 69% among women in ANZ, and 49% and 9% in men and women from Asia. During 2,906,013 person-years of follow up, there were 371 deaths from UADTC (176 lip, oral cavity or pharynx, 157 esophagus and 38 larynx; 74% in Asia; 87% male).

### 2.1. Current Smoking and Risk of Mortality from UADTC

**Figure 1.**
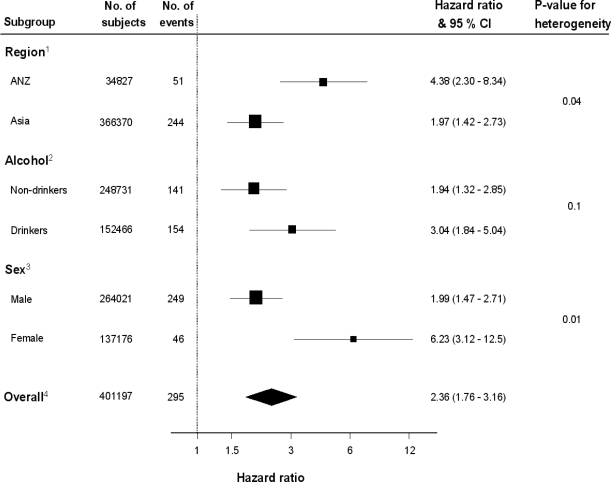
Hazard ratios for death due to upper aero digestive tract cancer for current smokers compared with those who have never smoked by subgroups and overall. Black square = point estimate (with area proportional to statistical ‘information’, based on inverse of variance of the HR estimated for each subgroup) and horizontal line = 95% CI for observed effect in each subgroup and overall. 1 = adjusted for age and alcohol (analyses from Cox models which were stratified by sex and study); 2 = adjusted for age (stratified by sex and study); 3 = adjusted for age and alcohol (stratified by study); 4 = adjusted for age and alcohol (stratified by sex and study)

Current smokers had more than twice the risk of mortality from UADTC, compared with never smokers: the HR was 2.36 (95% confidence interval, CI: 1.77 – 3.15), adjusted for age. The HR stayed the same after further adjustment for alcohol (Figure 1). For each subtype of UADTC, smoking was associated with a significant increase in the risk of mortality compared with never smoking. After adjusting for age and alcohol, the HR was: for lip, oral cavity or pharyngeal cancer, 2.14 (95% CI: 1.43 – 3.19); esophageal cancer, 2.84 (95% CI: 1.72 – 4.68); laryngeal cancer, 2.59 (95% CI: 1.07 – 4.68). The association between smoking and overall UADTC death was much stronger in ANZ compared with Asia, and in women compared with men (Figure 1).

### 2.2. Current Drinking and Smoking and UADTC

The HR for current drinking, compared with not, was 1.33 (95% CI: 1.03 – 1.73). However, after adjusting for current smoking, alcohol had very little association with the risk of mortality from UADTC: 1.01 (95% CI: 0.80 – 12.7). Current smoking had a significant effect among both drinkers and non-drinkers (Figure 1). Although the effect of smoking was considerably higher among those who consumed alcohol, there was no strong evidence of an interaction (p = 0.10), perhaps due to lack of information on the level of alcohol consumption.

### 2.3. Smoking and Risk of Mortality from UADTC in Former Smokers

Overall, 48,134 individuals (62% from Asia) reported at study baseline that they had quit smoking. During follow-up, a total of 70 (40% from Asia) former smokers died of UADTC. The HR for mortality from UADTC, comparing ex-smokers with current smokers, was 0.92 (95% CI: 0.66 – 1.27), adjusted for age and alcohol drinking. This HR differed by subtypes of UADTC: for lip, oral cavity or pharyngeal cancer, 0.99 (95% CI: 0.58 – 1.69); esophageal cancer, 1.03 (95% CI: 0.66 – 1.63); and laryngeal cancer, 0.33 (95% CI; 0.11 – 0.98).

### 2.4. Dose-Response Relationship between Current Smoking and UADTC

**Figure 2.**
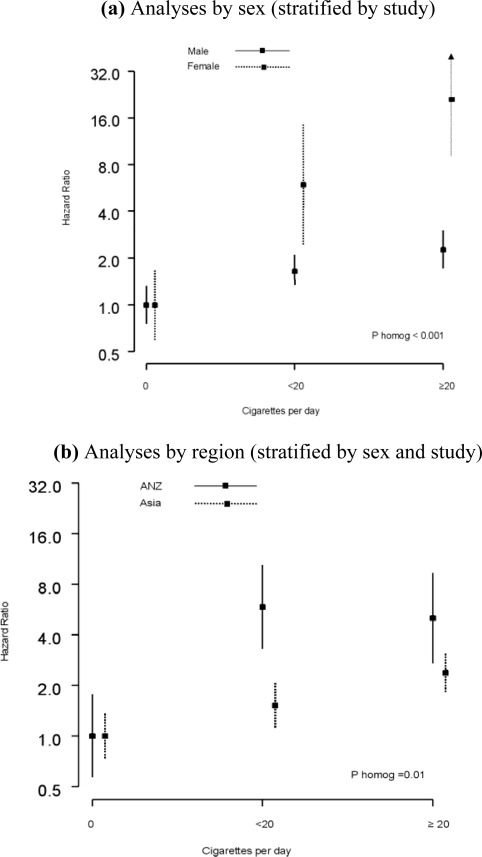
Hazard ratios for death due to upper aero digestive tract cancer for current smokers by number of cigarettes smoked per day, compared with those who have never smoked. Lines show 95% confidence limits; the arrow signifies that the upper end is off the scale. Hazard ratios are adjusted for age and alcohol.

Of the 26 studies, 17 reported the number of cigarettes smoked per day at baseline, involving 231,562 study participants. There was evidence of a dose-response association between the number of cigarettes smoked per day and mortality from UADTC and its subtypes (Table 2). Individuals who smoked more than 20 cigarettes a day had approximately three times the risk of dying from UADTC compared with never smokers. However, there was evidence of a sex difference in the strength of this association: the HR for smoking ≥ 20 cigarettes per day compared with never smoking was 2.27 (95% CI: 1.77 – 2.90) in men versus 21.1 (95% CI: 9.02 – 49.4) in women, after adjusting for age and alcohol; (Figure 2A). Similarly, significant differences were found between the regions (Figure 2B); the equivalent HRs were 5.03 (95% CI: 2.70 – 9.36) in ANZ versus 2.30 (95% CI: 1.77 – 2.99) in Asia.

### 2.5. Contribution of Smoking to Mortality from UADTC

**Figure 3.**
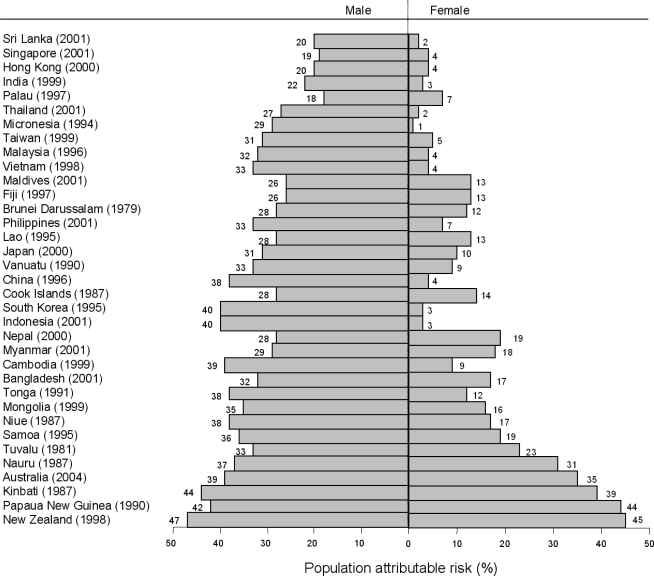
Population attributable risk (%) of upper aero digestive tract cancer for smoking. Data are derived from representative surveys (years of studies given in parentheses).

Figure 3 presents the population attributable fraction for males and females for UADTC caused by smoking for each country in the WHO Western Pacific and South-East Asian regions. The fraction of UADTC attributable to smoking ranged from 18–47% in males and from 1–45% in females.

### 2.6. Discussion

Analyses from this large dataset, with almost 400 deaths, confirm that smoking is strongly associated with the risk of mortality from UADTC in both Asia and ANZ. There was a significant dose-response association between the average number of cigarettes smoked daily and the risk for all subtypes of UADTC. Smoking had a stronger association with the risk of mortality from UADTC among female, compared with male, smokers. Although not statistically significant, the current study supports previous observations that concurrent alcohol consumption may exacerbate the effect of smoking on the risk of UADTC [7,14]: amongst non-drinkers, smoking doubled the risk of UADTC, whereas amongst drinkers it trebled the risk.

The findings of this study are consistent with earlier epidemiological evidence [15–23], but the estimated hazards are smaller than in some studies [18–23], especially those conducted in Western countries [20–23]. This may be due to differences in study design and the degree to which non-fatal events are included. But it may also signify a regional difference, since the Asian studies contributed 87% of participants, and 74% of events in APCSC, whilst the ANZ hazard ratio was significantly higher in this study. The lower excess risk for UADTC associated with smoking in Asia is likely to be due to the relative immaturity of the smoking epidemic across Asian countries [24], especially in China, where the habit only reached its peak in the mid-1990’s [25–29]. Other potential contributing factors to the smaller relative risks associated with smoking observed in the Asian cohorts are the greater exposure among non-smokers to indoor air-pollution from wood-burning in homes [30] combined with extremely high levels of passive smoking, particularly in China where an estimated 450 million individuals are so exposed [31].

The APCSC estimates suggest a greater risk of mortality from UADTC among female smokers compared with male smokers, in agreement with a recent study in Japan [32], but opposite to the finding of the Swedish Smoking Survey [33], although the latter was based on few events (11 in women and 33 in men). Several epidemiological studies have found a statistically significant increase risk for lung cancer among female smokers compared with male smokers, although others have found no difference [34–39]. It is currently unclear which mechanisms mediate the reported increased susceptibility among female smokers to cancers of the lung and upper-aero digestive tract. Potential explanations include sex differences in smoking behaviours (such as the degree of inhalation, for example) or differences in the accuracy of self-reporting of smoking consumption between men and women. Alternatively, a role for female sex hormones in increasing the susceptibility of women to tobacco carcinogens has been suggested [34–38]. Clearly, further research is required to determine whether the difference in risk between men and women is real and if so, what is driving it. Answers to these two questions could have repercussions for the development of sex-specific smoking cessation programs, particularly in countries where the prevalence of smoking is on the increase in young women [40].

In contrast with an overview of studies that reported a reduction in risk of UADTC among former smokers compared with current smokers of 30% to 50% in the first five years and up to 70% after 10 years of quitting smoking [41], data from the current study failed to demonstrate a significant reduction in risk of dying from UADTC among former smokers compared with current smokers, except for laryngeal cancer. We believe that our inability to find a protective effect of quitting overall is most likely an artifact of the current situation in Asia where quitting is unusual, and those who have quit will have abstained for a relatively short time. However, differences in the distribution of subsidiary site cancers within the overall definition of UADTC could also play a role.

The main strength of the current analysis is the large number of events, which facilitated our ability to assess associations in subgroups. However, we were limited by lack of information on duration of smoking and age of uptake of smoking, which may well be important determinants of the risk of smoking cigarettes for UADTC. Our data on alcohol drinking were also limited to baseline measures of current drinking and also to whether or not the subject drank; there is evidence [7,14] of a dose-response relationship between the amount of alcohol consumed and UADTC. Thus, we may have underestimated the true effect of drinking; there may be residual confounding due to our lack of data on the frequency of alcohol use. Furthermore, we had no data on changes in smoking and drinking status during follow-up. Although our analyses were restricted to deaths, the low five-year survival from UADTC [2–4], and the lack of systematic differences in excess risk found between previous studies that did [15,16,18,21–23] and did not [17,19,20] analyse non-fatal, as well as fatal, UADTC events, suggests that this has not introduced bias.

## 3. Conclusions

Findings from this study have clear implications for public health. For example, based on recent reports of the prevalence of smoking amongst men [10], and the effect of smoking found here, we estimate that 38% of male deaths from UADTC in China are attributable [13] to smoking. Effective tobacco control in the populous Asia Pacific region should prevent millions of deaths from UADTC, as well as other smoking-related outcomes, over the next few decades.

## Figures and Tables

**Table 1.** Characteristics at baseline and details of follow-up, for all studies, by region.

Country	Study	Baseline	No. of subjects	Mean age (yrs)	Female (%)	Current drinker s %		Current smokers (%)		Former smoker s (%)		Mean cigarettes per day		Media n FU (yrs)	UADT cancer deaths
						M	F	M	F	M	F	M	F		
Australia	Busselton	1966–81	7789	44.9	52	81	57	44	24	25	12	19	16	26.5	34
Australia	Long. Study of Aging	1992–93	1610	78.1	48	71	58	8	8	62	23	16	13	4.6	3
Australia	National Heart Foundation	1989–90	9277	43.5	51	87	75	27	21	32	20	21	16	8.3	12
Australia	Newcastle	1983–94	5929	51.7	50	85	65	28	18	37	18	20	18	8.9	13
Australia	Perth	1978–94	10230	45.0	48	90	75	30	21	31	18	20	16	14.4	9
Australia	WA AAA Screenees	1996–99	12203	72.2	0	82	-	11	-	60	-	14	-	3.2	22
	Fletcher	1992–94	10326	44.3	28	87	77	26	18	33	28	15	13	5.8	4
NZ	Challenge														
**ANZ**	**Subtotal**	**1966–99**	**57364**	**52.0**	**35**	**84**	**69**	**23**	**20**	**42**	**19**	**18**	**15**	**7.9**	**97**
China	Anzhen	1991	8378	53.8	55	39	3	51	10	9	2	15	10	4.3	5
China	Fangshan Guangzhou	1991–92	2619	47.3	67	43	2	75	22	6	2	15	9	3.6	1
China	Occupational Seven Cities	1985–98	166695	41.5	22	27	4	60	1	1	0	15	12	7.3	146
China	Cohorts	1987	10811	53.9	55	42	6	57	17	8	2	-	-	2.7	11
China	Yunnan	1992	6581	55.8	3	85	70	70	0	14	0	12	-	4.5	9
Hong Kong	Hong Kong	1985–91	2983	78.6	57	24	8	29	11	41	18	13	8	2.5	5
Japan	Akabane	1985–86	1834	54.5	56	62	5	62	1	21	0	23	9	11.0	1
Japan	Civil Service Workers	1990–92	9240	46.7	33	82	48	51	11	26	3	-	-	6.7	3
Japan	Hisayama	1961	1601	56.1	56	70	8	76	17	4	1	-	-	24.6	4
Japan	Konan	1987–95	1226	51.7	55	78	23	62	5	14	1	22	11	6.4	3
Japan	Ohasama	1992–93	2240	59.5	64	65	8	51	2	11	0	-	-	4.1	2
Japan	Saitama	1986–90	3615	54.5	62	77	33	63	8	21	2	22	13	11.0	9
Japan	Shibata	1977	2350	56.9	58	72	9	72	4	6	0	20	10	20.0	10
Japan	Shigaraki Town	1991–97	3730	57.1	59	70	19	59	8	22	2	22	13	4.4	4
Singapore	Singapore Heart	1982–97	2321	40.7	49	50	18	41	3	14	1	-	-	14.6	1
Singapore	Singapore NHS92	1992	3305	39.2	52	48	15	35	3	12	0	-	-	6.2	3
S. Korea	KMIC	1992	160242	44.0	33	73	9	58	0	21	0	-	-	4.0	49
Taiwan	CVDFACTS	1988–96	5729	47.2	55	16	1	48	1	7	0	-	-	6.0	2
Taiwan	Kinmen	1993–97	2545	63.2	49	44	4	50	5	17	1	-	-	2.9	6
**Asia**	**Subtotal**	**1961–98**	**398045**	**44.6**	**32**	**49**	**9**	**59**	**5**	**11**	**1**	**15**	**11**	**5.3**	**274**
**Total**		**1961–99**	**455409**	**46.0**	**32**	**53**	**17**	**55**	**7**	**14**	**3**	**16**	**14**	**5.9**	**371**

**Table 2.** Dose-response association between current cigarette smoking and upper aero digestive tract cancers (CPD=cigarettes currently smoked per day, where known). All analyses stratified by sex and study.

**Cancer type**	**No. of subjects**	**No. of deaths**	**Adjusted for age HR and 95 % CI**	**Adjusted for age & alcohol HR and 95 % CI**
**Lip, oral cavity or pharynx**				
Never-smoked	142700	57	1	1
<20 CPD	50077	34	1.83 (1.14–2.95)	1.86 (1.15–3.00)
≥20 CPD	38785	43	2.32 (1.47–3.65)	2.37 (1.49–3.78)
P for trend			<0.001	<0.001
**Esophagus**				
Never-smoked	142700	33	1	1
<20 CPD	50077	17	2.74 (1.37–5.49)	2.52 (1.25–5.07)
≥20 CPD	38785	25	3.89 (1.99–7.60)	3.40 (1.71–6.76)
P for trend			<0.001	<0.001
**Larynx**				
Never-smoked	142700	7	1	1
<20 CPD	50077	2	1.18 (0.22–6.17)	1.28 (0.24–6.76)
≥20 CPD	38785	9	5.25 (1.62–17.0)	5.91 (1.81–19.3)
P for trend			0.005	0.003
**Overall**				
Never-smoked	142700	97	1	1
<20 CPD	50077	53	2.03 (1.38–2.97)	2.01 (1.37–2.95)
≥20 CPD	38785	77	2.88 (2.01–4.11)	2.83 (1.96–4.09)
P for trend			<0.001	<0.001
